# Satisfaction guaranteed? How individual, partner, and relationship factors impact sexual satisfaction within partnerships

**DOI:** 10.1371/journal.pone.0172855

**Published:** 2017-02-23

**Authors:** Julia Velten, Jürgen Margraf

**Affiliations:** Mental Health Research and Treatment Center, Ruhr-Universität Bochum, Bochum, Germany; University of Ottawa, CANADA

## Abstract

Within committed relationships, a wide range of factors may challenge or facilitate sexual satisfaction. The aim of this study was to clarify which individual, partner-, and partnership-related aspects of a sexual relationship are crucial for the prediction of sexual satisfaction. The study included data of a representative sample of 964 couples from the general population. The actor-partner interdependence model was used to estimate actor and partner effects. Overall, predictors explained 57% of outcome variance. Actor effects were found for sexual function, sexual distress, frequency of sexual activity, desire discrepancy, sexual initiative, sexual communication, sociosexual orientation, masturbation, and life satisfaction. Gender-specific partner effects were found for sexual function and sexual distress. Neither age, nor relationship duration were significant predictors. To deepen our understanding of sexual satisfaction, it is necessary to take quantitative and qualitative aspects of sexual relationships into account and to consider actor-, partner-, and relationship-related predictors.

## Introduction

Countless myths surround sexual satisfaction. Mass media suggest that multiple or simultaneous orgasms, large penis size, or hour-long tantric sex are needed for women or men to feel sexually satisfied. While research has already falsified some of these myths (e.g., penis size) [1], and has pointed out a number of variables that are correlated to sexual satisfaction [2–4], the question of which factors *actually* matter when it comes to a satisfying sexual life still remains unanswered. Sexual satisfaction has been defined as the evaluation of positive and negative dimensions of one’s sexual relationship [5]. These dimensions may include personal experiences (e.g., how often one reaches orgasm during sex), the experiences of the sexual partner (e.g., how consistently a partner has an erection during sex), or relationship-related aspects of sexuality (e.g., how often a couple has sex or how openly sexual matters are discussed). An appropriate way to quantify which aspects of a sexual relationship contribute most to a satisfying sexual life is to consider a wide range of potential predictors, to use dyadic couples’ data, and to choose a data analysis technique that allows for estimation of the predictive value of actor-, partner-, and relationship-related factors [2,6,7].

### Dyadic approach to sexual satisfaction

Most studies that have investigated sexual satisfaction have done so with samples of individuals, not couples. However, in order to investigate sexual satisfaction, it is crucial to take into account the interpersonal context in which a substantial part of sexual activity happens [5]. Couples’ data enables researchers to use a dyadic data analysis approach that estimates actor effects (i.e., an actor’s own scores on predictor variables) and partner effects (i.e., a partner’s scores on predictor variables) on outcome variables such as sexual satisfaction [8]. Of the 197 studies on sexual satisfaction that were reviewed by Sanchez-Fuentes and colleagues [4], only 24 studies included non-clinical couples’ data and none of them used a sample selected to be representative for the general population. Most of the reviewed couples-studies focused on certain target-populations, for example pregnant women [9] or cancer survivors [10] and their partners, and investigated the impact of their specific life situation on sexual satisfaction. Others used relatively small, convenience samples [11,12]. Even though these studies may provide valuable insight into the relation between sexual satisfaction and, for example, coping with a serious medical condition, the generalizability of their findings is limited.

An additional short-coming of previous research on sexual satisfaction is that most predictors, such as sexual communication [11] or sexual function [13] have been examined in relative isolation, without taking other possible predictors into consideration [6].

To our knowledge, this is the first study to overcome limitations of previous studies that used convenience samples, restricted their sample to certain age groups or target populations or investigated certain predictor variables in isolation [6]. To investigate the relative significance of different of sexuality-related factors of sexual satisfaction, our study includes wide-ranging predictors, namely sexual function and distress, frequency of sexual activities alone or with a partner as well as sexual desire discrepancy, sexual communication, and sociosexual attitudes. These factors were selected to represent both quantitative and qualitative aspects of a sexual relationship, and to estimate the relevance of personal sexual attitudes and solitary sexual behaviors in comparison to factors that require a sexual partner. In addition to these sexuality related factors, we included life satisfaction to control for a more general well-being, and other predictors (i.e., passage of time, household income) were included to control for and to investigate their relevance for sexual satisfaction on an exploratory basis.

### Sexual function and sexual distress

Sexual function is positively correlated to sexual satisfaction in women and men [13–15]. Sexual desire, arousal, and orgasm consistency are associated with greater satisfaction [16]. Conversely, lack of vaginal lubrication, erectile dysfunction, early ejaculation, inability to reach orgasm, and pain during intercourse are associated with lower sexual satisfaction [13,17]. Moreover, preliminary evidence suggests that certain aspects of a partner’s sexual function (e.g., low sexual desire) may be also relevant for an actor’s sexual satisfaction [2]. Two of the most commonly used instruments for sexual function—the Female Sexual Function Index [18] and the International Index of Erectile Function [19]—have been criticized for not assessing personal distress related to sexual problems [20]. Assessing distress is crucial in identifying clinically relevant sexual dysfunctions [21] and in determining how sexual function (or the lack thereof) is experienced and evaluated by the individual.

### Sexual frequency and sexual communication

Greater frequency of sexual activity is related to greater sexual satisfaction in women and men [22]. The difference between the desired and the actual sexual frequency has been called sexual desire discrepancy [23]. Greater desire discrepancy is associated with lower relationship satisfaction [23], a variable that is strongly correlated to sexual satisfaction [24]. In another study, in which desire discrepancy was defined as the difference in desire between sexual partners, low discrepancy was positively related to both relationship and sexual satisfaction [25]. In dating partnerships, men tend to initiate sex almost twice as often as women [26]. Sexual satisfaction is associated with more frequent sexual initiation in both genders and less frequent negative response to a partner's initiation in women [26]. How the balance of sexual initiative between partners influences sexual satisfaction has not been established. However, Lau et al. [27] reported that the belief that the husband should always initiate sexual interactions was associated with lower sexual satisfaction in both spouses. Being able to openly express sexual wishes or to communicate sexual concerns with a partner is associated with greater sexual satisfaction [28]. In a study of 220 married couples, *indirectness* of communication about sexual intimacy was associated with lower sexual satisfaction [7].

### Sociosexual orientation and solitary sexual behavior

Sociosexual orientation has been described as the willingness to engage in sex outside of exclusive, committed relationships [29]. In a student sample, sociosexuality was *not* significantly correlated to sexual satisfaction [30]. It is, however, not yet established how sociosexual orientation influences sexual satisfaction within committed partnerships. It is not unlikely that over the course of long-term relationships, a positive attitude towards casual sex may contribute to more extramarital involvement and/or lower sexual satisfaction within the partnership. The effects of solitary sexual behavior on sexual satisfaction within partnerships may be diverse. Women that had never reached orgasm through self-stimulation reported lower sexual satisfaction compared to women who had had this experience [31]. More frequent masturbation was, however, related to *lower* sexual satisfaction in women and men [32].

### Other potential predictors

Passage of time (i.e., age or relationship duration) is negatively correlated to sexual frequency [33] and older participants tend to have lower levels of sexual function [34]. The relationship between passage of time and sexual satisfaction may be less pronounced. However, Liu [35] reported a small and negative effect of relationship duration on the perceived quality of marital sex. The effect of the duration of a relationship on sexual satisfaction may also be gender-specific. Compared to men, women's sexual satisfaction may be more strongly related to relationship duration [36]. To investigate passage of time in unison with other sexuality-related predictors may be useful to clarify its relative contribution to a satisfying sexual life.

Satisfaction with life has been defined "as a global assessment of a person's quality of life" [37, p. 478]. Young, married individuals reported greater levels of life satisfaction compared to their unmarried counterparts [38]. Life satisfaction is related to other aspects of general well-being [39], and may also be associated to sexual satisfaction. Using factor-analysis, Fugl-Meyer et al. [40] found that sexual satisfaction was closely related to satisfaction in other areas of life, namely satisfaction with the ability to care for themselves, as well as satisfaction with partnership and family life. Taken together, these facets of satisfaction explained more than 50% of general life satisfaction.

Few studies have been conducted to assess the influence of socioeconomic factors on sexual satisfaction [6]. In one study by Rainer and Smith [41], household income was unrelated to sexual satisfaction. In another study, income was negatively related to sexual satisfaction in men [42]. The authors offer two possible explanations for these findings: Men with high income might have more access to extramarital affairs or might have extraordinarily high expectations for their sexual fulfillment. Both factors may lower satisfaction within their current partnerships. However, higher socioeconomic status is also associated with better mental and physical health [43,44], which are correlated with higher sexual function [45], a variable closely related to sexual satisfaction. To date, there is no consensus on how household income as well as an individual’s personal contribution to this income may relate to sexual satisfaction.

The aim of the present study was to investigate which aspects of sexual relationships contribute most to sexual satisfaction. Based on the findings summarized above, we hypothesized the following actor effects: Sexual function and sexual frequency would be positive predictors of sexual satisfaction; whereas sexual distress, desire discrepancy, sociosexual orientation and masturbation would be negatively predictive of sexual satisfaction. We further hypothesized that sexual communication would have a positive impact on actors’ sexual satisfaction, and that, taken together with the other predictors, passage of time (i.e., relationship duration and age) would not be predictive of actors’ sexual satisfaction. Because previous evidence was limited, partner effects were only hypothesized for sexual function―we expected that high sexual functioning in partners would be associated with greater actor sexual satisfaction. Additional predictors (e.g., sexual initiative, life satisfaction, income, and proportion of own income) were included on an exploratory basis.

## Methods

### Participants

In total, 964 couples (*N* = 1928 individuals) completed a survey about relationships and sexuality. Table 1 gives an overview of the sample characteristics.

**Table 1:** Sample characteristics.

		Complete sample
		(*N* = 1928)
		*M (SD)*
Age (Range: 18–90)		51.28 (12.73)
Partnership duration (in years; Range: 0–66)		23.98 (13.79)
Children (Range: 0–8)		1.70 (1.14)
		*n*^a^ (%)
Marital status		
	Married	1674 (87.5)
	Civil union	21 (1.1)
	Single	140 (7.3)
	Other (e.g., divorced, widowed)	93 (4.1)
Co-habitation with a partner		
	Yes	1855 (96.2)
	No	73 (3.8)
Household income per month in Euro		
	< 2,000	273 (15.2)
	2,000–3,000	470 (26.2)
	3,000–4,000	422 (23.5)
	> 4,000	628 (35.0)
Education level		
	No high-school degree	625 (32.4)
	High-School degree	208 (10.8)
	College degree	1095 (56.8)
Occupation		
	Full-time occupation	933 (49.0)
	Part-time occupation	367 (19.3)
	Retired	375 (19.7)
	Housewife/House husband	130 (6.8)
	Other	123 (6.7)

^a^ Numbers vary due to missing data.

In our sample, men were significantly older than women, *t*(1922) = -4.72, *p* < .001. Significant gender differences were also found for college education, χ^2^ = 53.34 (2), *p* < .001, with more men than women having a college degree; and occupation, χ^2^ = 495.71 (4), *p* < .001, with men more likely to be working full-time or retired. Ninety-eight percent (*n* = 950) of the couples were in a heterosexual relationship. The remaining two percent included 9 (0.9%) male-male and 5 (0.5%) female-female couples.

### Procedure

Computer-assisted telephone interviews were conducted for screening purposes and to gather participants' informed consent. The study aimed to include a representative sample of the German adult population. To accomplish representativeness, the sample was drawn from the residential population aged 18 years and above that was accessible via landline or mobile phones. Landline telephone numbers were chosen based on regional stratification while mobile phone numbers were stratified by providers. A within household random-sampling technique was used to facilitate random selection of individuals and to minimize sampling bias.

During the telephone screening, it was assessed if the respective household member was in a steady relationship. If the person answered affirmatively, the interviewer asked if he or she would be willing to participate in a study on relationship factors and sexuality together with his or her partner. After receiving detailed information about the survey, informed consent of both partners was obtained verbally. Participants were insured that they could withdraw their consent at any given point (i.e., by not returning their questionnaires or by not finishing their online survey) without negative consequences. To increase response rates and ensure representativeness of the sample, written consent was not obtained. Individuals without a steady partner were also eligible and received a modified version of the questionnaire. Their data will be presented elsewhere. All participants could choose to participate via online or paper-pencil survey. Study information (e.g., content, duration, and voluntariness) was again presented on the first page of the survey. The Ethics Committee of the Faculty of Psychology at the authors’ university approved the study.

Participants were informed not to discuss any content of the study before both partners had completed and submitted their surveys. A maximum of three reminder calls or emails were made or sent to increase response rates. The study was conducted from September 2015 to January 2016. Of 8,153 identified target persons, 3,467 individuals (42.5%) gave their informed consent to participate. Of all 2275 couples that agreed to participate, 964 (42.4%) returned questionnaires from both partners. A total of 1144 (59.2%) individuals participated online, the remaining participants chose paper-pencil format.

### Measures

#### Sexual function

Female sexual function was assessed with the Female Sexual Function Index (FSFI) [18], a self-report scale that measures sexual functioning over the previous four weeks. Items are answered on a scale from 1 to 5, with higher scores indicating better sexual function. Some questions include the additional answer category of 0, indicating no sexual activity during the last month. Items can be combined into one total score, ranging from 1.2 to 36 points (To allow for a calculation of the total score of women that had missing values, we calculated the mean scores of all subscales before weighting them according to the instructions by Rosen et al. [18]. This led to a change of the total range from 2–36 to 1.2–36. This procedure had no impact on the results of this study.) with a clinical cutoff of 26.55 [46]; women scoring below that cutoff are deemed at risk for sexual dysfunction. The validation of the German FSFI yielded good psychometric properties [47]. In the present study, internal consistency for the total scale was excellent with Cronbach's α = .97. Male sexual function was assessed with the International Index of Erectile Dysfunction (IIEF) [19]. Items are answered on a scale from 0 to 5, with higher scores indicating better sexual function. A total score can be calculated, ranging from 5 to 75. In a German validation study of the IIEF, a value of 53 for the total scale was the appropriate cutoff score to identify men with erectile dysfunction [48]. The psychometric properties of the IIEF have been approved in various populations and language versions [49]. In this study, internal consistency was excellent with Cronbach's α = .91.

#### Sexual satisfaction and sexual distress

Two single item measures were used to assess the degree to which participants were satisfied with their sexual life as well as how much they were distressed by their own sexual problems. Both items were answered on a scale ranging from 0 to 100, with lower scores indicating lower satisfaction or lower distress, respectively. Single item measures are frequently used to assess sexual satisfaction in individuals and couples [6].

#### Sexual frequency and sexual discrepancy

The frequency of sexual activities with a partner and frequency of masturbation were assessed on a 6-point scale with answer categories never, less than once a month, once to three times a month, once to twice a week, three to four times a week, and five times a week and more. All participants were asked how often they were currently having sex with their partner, alone or by themselves, and how frequently they desired partner-sex. The difference between the actual sexual frequency and the desired frequency was calculated in order to estimate how well the current situation reflects their personal preference. This variable was coded on a 4-point scale ranging from 0 to 3, with lower scores indicating lower discrepancy between actual and desired frequency of sexual activity. Only some participants (*n* = 62, 3.3%) indicated they would prefer having sex less often, 794 participants (43.1%) were satisfied with the actual frequency, and 985 (52.6%) wished for more sex than they had. Hence, a greater sexual discrepancy in most cases reflect a desire to have *more frequent* sex.

#### Sexual communication

The perceived quality of the sexual communication between two primary partners was assessed with a short version of the Dyadic Sexual Communication Scale (DSCS) [50]. The DSCS has been validated in population-based studies [51]. The scale measures with six Likert-style items, answered on a scale from 1 (*disagree strongly*) to 6 (*agree strongly*), how individuals perceive the discussion of sexual matters with their partner. In the present study, internal consistency was acceptable with Cronbach's α = .75.

#### Satisfaction with life

Life satisfaction was assessed with the Satisfaction With Life Scale [52], a five item questionnaire designed to assess the judgmental component of personal wellbeing rated on a scale ranging from 1 (*strongly disagree*) to 7 (*strongly agree*). Summing across items yields a total score ranging from 5 to 35, with a cut-off of 19 for at least average life satisfaction. The SWLS exhibits excellent psychometric properties [53]. In this study, Cronbach’s α was .90.

#### Passage of time

To assess two aspects related to passage of time, relationship duration and age in years were both assessed and included in the analysis.

#### Socioeconomic factors

Household income per year was assessed in order to estimate the socioeconomic status of the couple. Additionally, both partners were asked about the percentage of money that they personally contributed to the household income. This variable was added to estimate the distribution of roles (e.g., main income earner, homemaker) within the partnership.

### Data analysis

**Fig 1:**
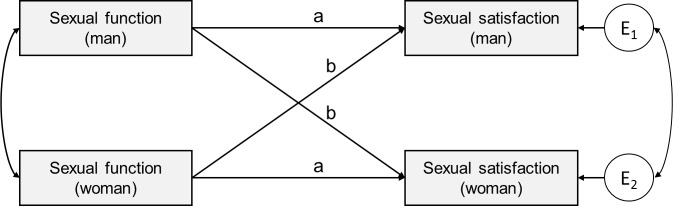
Actor–Partner Interdependence Model (APIM). The boxes on the left indicate the independent variables for men and women, the boxes on the right indicate the dependent variable for each. E1 and E2 represent the residual error of sexual satisfaction for men and women, respectively. Single-headed arrows indicate predictive paths. Double-headed arrows indicate correlated variables. Paths labeled with ‘a’ indicate actor effects, and paths labeled with ‘b’ indicate partner effects.

An Actor-Partner Independence Model (APIM) was calculated to simultaneously estimate actor and partner effects on the actor’s sexual satisfaction [8,54]. An important advantage of APIM is that it accounts for the nonindependence of responses of the two individuals that are involved in a dyadic relationship. Thereby, it allows researchers to investigate the interdependence between two individuals in a couple and includes the appropriate statistical methods for testing it. The APIM has been increasingly applied to and recommended for the study of close relationships [55]. Fig 1 shows an example of an APIM for sexual function as predictor of sexual satisfaction. See S1 Text for the multilevel formula used to predict actor’s sexual satisfaction.

Data were analyzed with an R based [56] online application (available at: https://davidakenny.shinyapps.io/APIM_MM) [57]. The APIM analysis uses generalized least squares analysis with correlated errors and restricted maximum likelihood estimation. The tests of coefficients within the APIM analysis were Z-tests and the tests of correlations were based on t-tests of correlation coefficients. All predictors were grand-mean centered before the analysis. The partial correlations between predictor and outcome variables, controlling for all other predictors, were calculated as effect sizes. Values above *r* = .10 indicate a small, above *r* = .30 a medium, and values above *r* = .50 a large effect size [58].

## Results

### Descriptive analyses

Sexual satisfaction was greater in women compared to men and in younger compared to older participants. Men reported greater distress due to their own sexual problems than women and highest distress ratings were reported by the oldest age group. Sexual function—assessed with gender specific instruments—differed significantly between age groups with younger participants indicating higher levels of sexual function. Sexual communication between partners was rated higher by women and younger participants. Life satisfaction was greater in women and in older participants. Table 2 summarizes these findings.

**Table 2:** Sample characteristics for sexuality-related variables by gender and age.

			Gender					Age								
	Complete sample		Women		Men		*p*	18–35 years		36–50 years		51–60 years		61 years and more		*p*
	(*N* = 1928)		(*n* = 960)		(*n* = 968)			(*n* = 244)		(*n* = 661)		(*n* = 572)		(*n* = 447)		
	*M*	*SD*	*M*	*SD*	*M*	*SD*		*M*	*SD*	*M*	*SD*	*M*	*SD*	*M*	*SD*	
Sexual satisfaction	67.80	25.99	70.08	26.08	65.57	25.71	< .001	72.95	22.87	68.61	25.32	68.70	25.81	62.45	28.07	< .001
(Range: 0–100)																
Sexual distress	27.26	26.73	25.68	27.37	28.80	25.98	.012	23.05	25.58	24.38	25.98	28.65	27.72	32.22	26.43	< .001
(Range: 0–100)																
Female sexual function			24.18	9.77				27.47	6.62	25.69	9.12	24.07	9.90	18.93	10.80	< .001
(Range: 2–36)																
Male sexual function					54.92	18.35		64.36	9.42	58.72	16.04	55.42	17.63	44.97	20.75	< .001
(Range: 5–75)																
Sexual communication	4.15	1.13	4.27	1.12	4.04	1.13	< .001	4.53	1.04	4.24	1.11	4.11	1.12	3.86	1.14	< .001
(Range: 0–6)																
Life satisfaction	5.34	1.16	5.39	1.16	5.29	1.16	.047	5.34	1.10	5.23	1.21	5.36	1.12	5.48	1.11	.007
(Range: 1–7)																

Male and younger participants reported more frequent masturbation. Men and younger participants indicated a higher desired frequency of sexual interactions than women and older individuals. Table 3 presents an overview of the sexuality-related frequency variables. See S1 Table for the zero-order correlations of all predictor and outcome variables and S1 Fig for a graphical display of the relationship between standardized predictor variables and sexual satisfaction.

**Table 3:** Frequency of solitary and partner-related sexual activities by gender and age.

				Gender					Age								
		Complete sample		Women		Men		*p*	18–35 years		36–50 years		51–60 years		61 years and more		*p*
		(*N* = 1928)		(*n* = 960)		(*n* = 968)			(*n* = 244)		(*n* = 661)		(*n* = 572)		(*n* = 447)		
Masturbation		*n*^a^	*%*	*n*^a^	*%*	*n*^a^	*%*	< .001	*n*^a^	*%*	*n*^a^	*%*	*n*^a^	*%*	*n*^a^	*%*	< .001
	Never	427	23.2	291	31.8	136	14.7		44	18.4	112	17.7	117	21.3	153	36.7	
	Less than once a month	481	26.1	307	33.5	174	18.8		42	17.6	158	24.9	162	29.5	118	28.3	
	1–3 times a month	434	23.5	208	22.7	226	24.4		57	23.8	148	23.3	139	25.3	89	21.3	
	1–2 times a week	288	15.6	74	8.1	214	23.1		50	20.9	116	18.3	81	14.7	40	9.6	
	3–4 times a week	142	7.7	29	3.2	113	12.2		27	11.3	65	10.3	37	6.7	13	3.1	
	5 times a week or more	72	3.9	7	0.8	65	7		19	7.9	35	5.5	14	2.5	4	1	
Frequency of sexual activity (actual)								.629									< .001
	Never	159	8.5	84	9.0	75	7.9		3	1.2	20	3.1	42	7.6	94	21.8	
	Less than once a month	253	13.5	121	13.0	132	14.0		15	6.2	98	15.2	78	14.1	59	13.7	
	1–3 times a month	649	34.6	326	35.1	323	34.1		79	32.5	223	34.6	204	36.9	143	33.2	
	1–2 times a week	617	32.9	309	33.2	308	32.6		99	40.7	230	35.7	180	32.5	108	25.1	
	3–4 times a week	152	8.1	72	7.7	80	8.5		40	16.5	60	9.3	34	6.1	17	3.9	
	5 times a week or more	46	2.5	84	1.9	28	3.0		7	2.9	14	2.2	15	2.7	10	2.3	
Frequency of sexual activity (desired)								< .001									< .001
	Never	47	2.5	34	3.7	13	1.4		1	0.4	9	1.4	11	2.0	26	6.2	
	Less than once a month	65	3.5	50	5.5	15	1.6		3	1.2	14	2.2	18	3.3	28	6.6	
	1–3 times a month	433	23.3	284	31.0	149	15.9		27	11.2	121	18.9	149	27.2	135	32.0	
	1–2 times a week	815	43.9	394	43.0	421	44.8		97	40.1	298	46.5	248	45.3	172	40.8	
	3–4 times a week	379	20.4	133	14.5	246	26.2		88	36.4	153	23.9	94	17.2	43	10.2	
	5 times a week or more	118	6.4	22	2.4	96	10.2		26	10.7	46	7.2	28	5.1	18	4.3	

^a^ Numbers vary due to missing data.

### Actor-partner-interdependence model

Gender makes a meaningful difference in the prediction of sexual satisfaction, as was indicated by a significant test of overall distinguishability, χ2 = 34.24 (21), *p* = .012. Hence, separate actor and partner effects were estimated for women and men. For the APIM analysis, a total of 731 dyads with complete data were included. The amount of variance explained by the full model was R^2^ = .55 for women and R^2^ = .60 for men (R^2^ = .57 in total). The bivariate correlation between the two partner's scores on sexual satisfaction was *r* = .57, *p* < .001, the partial correlation controlling for all predictors was *r* = .25, *p* < .001. Of the total non-independence in sexual satisfaction between partners, 53.7% could be explained by the APIM and 27.8% by the between-dyads covariates. Table 4 shows the results for the APIM for sexual satisfaction for women and men. Please see S2 Table for the summary of the APIM analysis across genders.

**Table 4:** Actor-partner interdependence model for sexual satisfaction in women and men.

Variable	Role	Effect	Estimate	CI (95%)	*p*	β	*r*
Sexual satisfaction	women	Intercept	68.71	66.67–70.75	< .001		
	men	Intercept	71.55	69.74–73.37	< .001		
Sexual function	women	Actor	8.93	6.79–11.08	< .001	.32	.30
		Partner	-1.18	-3.39–1.04	.299	-.04	-.04
	men	Actor	6.60	4.45–8.74	< .001	.24	.24
		Partner	2.99	0.78–5.21	.001	.11	.12
Sexual distress	women	Actor	-17	-0.22–-0.11	< .001	-.18	-.21
		Partner	-0.10	-0.15–-0.04	< .001	-.11	-.14
	men	Actor	-0.14	-0.20–-0.08	< .001	-.15	-.21
		Partner	-0.04	-0.09–0.02	.159	-.04	-.06
Frequency of sexual activity	women		3.98	2.29–5.68	< .001	.16	.19
	men		0.47	-1.10–2.04	.556	.02	.04
Desire discrepancy	women	Actor	-5.88	-8.08–-3.67	< .001	-.18	-.19
		Partner	-0.56	-2.48–1.37	.572	-.02	-.02
	men	Actor	-5.55	-7.76–-3.34	< .001	-.17	-.21
		Partner	-3.87	-5.80–-1.95	< .001	-.12	-.15
Sexual initiation	women		-0.94	-3.17–1.30	.412	-.02	-.03
	men		-4.24	-6.10–-2.38	< .001	-.11	-.16
Sexual communication	women		2.60	1.31–3.89	< .001	.12	.15
	men		3.84	2.66–5.02	< .001	.17	.24
Sociosexual orientation	women	Actor	-3.12	-5.20–-1.05	.003	-.10	-.12
		Partner	0.43	-1.42–2.28	.648	.01	.02
	men	Actor	-3.17	-5.24–-1.10	< .001	-.10	-.14
		Partner	0.03	-1.82–1.88	.975	.00	.00
Masturbation	women	Actor	-1.50	-2.83–-0.18	.026	-.08	-.09
		Partner	-0.39	-1.46–0.67	.469	-.02	-.03
	men	Actor	-1.23	-2.56–0.09	.011	-.07	-.09
		Partner	0.92	-0.15–1.98	.117	.05	.06
Age	women	Actor	0.16	-0.12–0.44	.259	.08	.05
		Partner	-0.21	-0.47–0.06	.124	-.10	-.06
	men	Actor	0.07	-0.22–0.35	.589	.03	.02
		Partner	-0.15	-0.41–0.11	.234	-.08	-.04
Relationship duration	women		0.12	-0.04–0.28	.152	.06	.05
	men		0.08	-0.07–0.23	.291	.04	.05
Life satisfaction	women	Actor	2.18	0.90–3.46	< .001	.10	.13
		Partner	0.39	-0.88–1.65	.548	.02	.02
	men	Actor	2.22	0.94–3.50	< .001	.10	.14
		Partner	1.05	-0.21–2.32	.071	.05	.07
Household income	women		-0.66	-1.23–-0.09	.023	-.06	-.10
	men		-0.58	-1.12–-0.03	.039	-.06	-.08
% of household income	women	Actor	0.08	0.00–0.16	.043	.07	.08
		Partner	0.06	-0.02–0.14	.144	.06	.06
	men	Actor	-0.01	-0.09–0.07	.849	.01	-.01
		Partner	0.05	-0.04–0.13	.182	.04	.05

#### Actor effects

The following significant actor effects were found: In both women and men, sexual function and life satisfaction were positively predictive of sexual satisfaction; while sexual distress, desire discrepancy, sociosexual orientation, and masturbation were negatively predictive of sexual satisfaction. Furthermore, the percentage of household income earned by the female partner was a positive predictor of women’s, but not men’s sexual satisfaction. With respect to the between-dyads variables (i.e., all variables that had only one value per couple such as relationship duration), sexual communication was a positive and household income was a negative predictor in both genders. Frequency of sexual activity was a positive predictor in women, meaning that greater sexual frequency was associated with greater sexual satisfaction in women. Sexual initiative was a negative predictor in men, indicating that a balanced sexual initiative was associated with greater sexual satisfaction in men.

#### Partner-effects

For sexual function, the partner effect from women to men was statistically significant, indicating that the greater the sexual function of a man’s partner, the greater his sexual satisfaction was. For sexual distress, the partner effect from men to women was statistically significant, indicating that sexual distress of a male partner was associated with lower sexual satisfaction in the female. For desire discrepancy, the partner effect from women to men was significant. Men whose partners indicated greater desire discrepancy reported lower sexual satisfaction.

#### Actor-partner interaction effects

The actor-partner interaction effect for sexual function was significant for both women and men (*p* < .001). The partner effect for actors who had high sexual function (one SD above mean) was 6.63 (*p* < .001) and for actors who had low sexual function (one SD below mean) was 0.18 (*p* = .794). This indicates that a partner’s sexual function was only a significant predictor of sexual satisfaction for individuals whose own sexual function levels were high. For women, the actor-partner interaction for desire discrepancy was statistically significant (*p* = .002). The partner effect for women, who reported high desire discrepancy (one SD above mean), was -2.35 (*p* = .046) and for women who reported low desire discrepancy (one SD below mean), the effect equaled 2.01 (*p* = .086). This indicates that the effect of a partner’s desire discrepancy depends on the level of desire discrepancy that the woman experiences herself.

## Discussion

The main objective of our study was to demystify sexual satisfaction and to clarify which aspects of a sexual relationship contribute most to satisfying sexual lives within partnerships. It is the first study that used a population-based, representative sample of couples of all age groups and a wide range of sexuality-related predictors that could explain 57% of outcome variance.

In line with our hypotheses, actor sexual function was the strongest positive predictor of sexual satisfaction in our model. Interestingly, the proposed partner effect of sexual function was significant only in men. Women's sexual function, which includes, for example, how consistently she reaches climax, was also important for men's sexual satisfaction. Actor's distress by own sexual problems was a negative predictor for both women and men. Partner distress was, however, only predictive in women; that is, women who have a partner that is distressed by a sexual problem have lower sexual satisfaction. Taken together, these findings suggest that women and men respond to different aspects of a partner's sexual functioning: Men are more likely to be negatively affected by women's lack of sexual function in the narrower sense (e.g., their lack of arousal), while women are more affected by a partner's distress about a perceived sexual problem, not necessarily the lack of function (e.g., the erectile problem) itself. This finding is in line with previous research that proposed that certain aspects of a partner’s sexual functioning may indeed be related to an actor’s sexual functioning [11]. In addition to this, the significant actor-partner interaction for sexual function indicates that a partner's sexual function is only predictive of an actor's satisfaction in individuals that have an above average sexual function level. In other words, in individuals with sexual problems, sexual satisfaction is independent of their partner's function. A minimum level of sexual function may be needed to consider a partner's sexual function as relevant for one’s own evaluation of a satisfying sexual life. Future studies may clarify which aspects of a partners’ sexuality impact sexual satisfaction in women and men, and under which circumstances.

In contrast to our hypothesis, frequency of sexual activity was only a significant predictor in women, but not in men. This finding partially contradicts another study that found positive effects of sexual frequency on both husbands’ and wives’ sexual satisfaction [22]. However, Schoenfeld et al. focused on non-sexual behaviors as predictors for sexual and marital satisfaction and did not include other sexuality-related predictors such as sexual initiative, which, in our study, was a negative predictor only in men, and not in women. This means that men’s sexual satisfaction was highest, when sexual activity was initiated equally often by both partners.

Before drawing further conclusions, it makes sense to take a third predictor, sexual desire discrepancy—the difference between actual and desired frequency of sex—into account. Desire discrepancy was a negative predictor in both genders. This finding partially contradicts previous studies, which have suggested that sexual desire discrepancy may actually be more important for women’s sexual satisfaction when controlling for relationship satisfaction, than for men’s [59].

Taken together, this pattern of results may be explained by the interdependencies of these three variables within sexual relationships. Men generally initiate sex more often and desire a higher frequency of sex than women [26]. In non-abusive relationships, sexual activities require the consent of both partners. Thereby, the member of the couple that desires sex less frequently—which is more likely to be the female partner—is often the “limiting factor” that determines how frequently a couple has sex [60]. Hence, men may more often experience a negative response to their sexual initiatives. A female partner that initiates sex might increase a man's feeling of desirability and reduce the negative affect associated with sexual rejection. For women, it may be less relevant who initiates sex. The total frequency of partnered sex—which includes her sexual initiations as well as her positive responses to his initiatives—is the more important predictor of her sexual satisfaction. Finally, men, on average, tend to “get” sex less frequently than they desire and this may partly explain the lower levels of sexual satisfaction reported by men in our study.

In addition to these rather *quantitative* indicators of sexual behavior, sexual communication was a positive predictor in both women and men. To talk openly about sexual wishes and preferences, but also about issues or problems, might be especially relevant to increase the *quality* of sexual interactions within a partnership [28].

As hypothesized, actors’ sociosexual orientation and frequency of masturbation were negatively predictive of sexual satisfaction in both genders. A positive attitude towards casual, uncommitted sex is associated with more sexual partners [29]. More sexual experiences may lead to increased sexual expectations and might result in lower satisfaction with the status quo of the sexual life within a long-term partnership. Although masturbation is no longer socially condemned and some authors even see it as a means to increase sexual health [61], within partnerships, masturbation might be used to compensate for a lack of partnered sex and may still be associated with negative affects such as guilt or shame [62].

General life satisfaction was a positive predictor of sexual satisfaction. This finding corresponds well to previous studies that have identified sexual satisfaction and general life satisfaction as closely related [40]. Due to the cross-sectional nature of these studies, the direction of effects cannot be clarified. Also, future studies should investigate if this finding is mediated by a more general relationship satisfaction or if there is a direct association between sexual satisfaction and the judgmental component of general wellbeing.

Household income was negatively predictive of sexual satisfaction in both genders, although bivariate correlations between the two variables were not significant. Taking a closer look at the correlations with other predictor variables (see S1 Table), on the one hand, income was positively correlated with higher sexual function as well as life satisfaction, which are two positive predictors of sexual satisfaction. On the other hand, income was also correlated to masturbation and a more casual sociosexual orientation, two variables that predicted lower sexual satisfaction. Although our finding is in line with previous studies [6], more research is needed to clarify which variables (e.g., sex-related attitudes such as sociosexuality or conservatism, physical and mental health) might mediate or moderate the relationship between income and sexual satisfaction.

Another interesting finding is that the proportion of household income that is earned by the female has a small, but significant, positive impact on women's sexual satisfaction. A more equal distribution of work, and thereby possibly power, within a relationship may facilitate women's, but not men’s, sexual satisfaction [63].

Another aim of this study was to provide general population estimates for factors that characterize the sexual relationships of couples. As expected, sexual function and frequency were highest in the youngest and lowest in the oldest participant group [33]. However, the majority of older participants (age 61 and older) were still sexually active with 62% engaging in sexual activity at least once a month. Sexual satisfaction was also lowest in our oldest participant group. In contrast to other studies [e.g. 35], this decline was not predicted by age or relationship duration, but fully mediated by other predictors. Decade-long marriages or old age do not diminish sexual satisfaction by themselves. To improve sexual communication and to (re-)establish a regular sexual routine may be ways to foster a satisfying sexual life, regardless of age. Overall, our findings underline the great extent of variability in sexual relationships that exists across as well as within age groups and genders. Simplistic generalizations about what sexual behavior constitute “normal” or “healthy” sexual relationships should be treated with skepticism.

Several limitations challenge the internal validity and generalizability of our findings. The volunteer bias that is known in sexuality related research may have been particularly relevant for our study [64]. Although our sample was selected to be representative for the general population, people with more conservative sexual attitudes may have felt uncomfortable with the study's topic and thus have been unlikely to participate. Our study required the consent of both partners to participate. Couples with relationship discord are therefore most likely underrepresented in our study. The use of a single item measure of sexual satisfaction is not without problems. Although similar items have been used in numerous studies [6] and have face validity, the use of a well-validated questionnaire would have increased the reliability of our results. In this study, predicting variables were selected to cover a broad range of factors that characterize solitary and partner-related sexual behaviors in partnerships. However, as the predictors were not based on theoretical grounds, our findings could be spurious. Other variables not assessed in this study may drive both predictors and our outcome variable.

Future studies should include longitudinal data to examine a possible bidirectional relationship between the predictor variables and sexual satisfaction. Our findings also have clinical implications for the diagnosis and treatment of sexual difficulties. Clinicians are encouraged to inquire about both quantitative (e.g., frequency of sexual activity) and qualitative aspects (e.g., sexual communication) of a sexual relationship as well as a partner’s sexual function when exploring sexual concerns. They may also encourage patients to explicitly work on improving sexual communication with their partners as a positive communication about sexual wishes and concerns may facilitate sexual satisfaction, even in the presence of a sexual dysfunction (e.g. an erectile disorder).

### Conclusion

Sexual satisfaction has often been investigated in the context of sexual health objectives or marital satisfaction. However, to find out which factors facilitate a fulfilling sexual life is also an important research objective on its own. Sexual satisfaction within steady partnerships was influenced by different actor-, partner-, and relationship-related factors, which together explained 57% of the outcome variance. Actor and partner sexual function, sexual communication, and actor's life satisfaction were positive predictors. Actor's and partner's sexual distress, actor's sexual desire discrepancy, sociosexual orientation, masturbation, and household income were negative predictors. Some gender specific patterns emerged: Sexual frequency was a positive predictor only in women. A balance of sexual initiative between partners was a positive predictor of men's sexual satisfaction. Taking all other predictors into account, age and relationship duration did not predict sexual satisfaction. Our study supports the notion that sexual satisfaction is multi-determined and is best explained by considering actor, partner, and interpersonal dimensions of sexual relationships.

## Supporting information

S1 Dataset (SAV)

S1 Fig Relationship between standardized predictor variables and sexual satisfaction. (TIF)

S1 Table Bivariate correlations between the actor-, partner-, relationship-related predictors, and sexual satisfaction. (DOCX)

S2 Table Overall effect estimates for the actor-partner interdependence model for sexual satisfaction. (DOCX)

S1 Text Multilevel formula for the prediction of sexual satisfaction. (DOCX)

## References

[1] Lever J, Frederick D, Peplau LA. Does size matter? Men’s and women’s views on penis size across the lifespan. Psychol Men Masc. 2006;7: 129–143.

[2] Yucel D, Gassanov MA. Exploring actor and partner correlates of sexual satisfaction among married couples. Soc Sci Res. 2010;39: 725–738.

[3] Štulhofer A, Ferreira LC, Landripet I. Emotional intimacy, sexual desire, and sexual satisfaction among partnered heterosexual men. Sex Relatsh Ther. 2014;29: 229–244.

[4] Schmiedeberg C, Schröder J. Does sexual satisfaction change with relationship duration? Arch Sex Behav. 2015;45: 1–9. 10.1007/s10508-015-0587-0 26246315

[5] Lawrance KA, Byers ES. Sexual satisfaction in long-term heterosexual relationships: The Interpersonal Exchange Model of Sexual Satisfaction. Pers Relationship. 1995;2: 267–285.

[6] Sanchez-Fuentes MM, Santos-Iglesias P, Sierra JC. A systematic review of sexual satisfaction. Int J Clin Heal Psychol. 2014;14: 67–75.

[7] Theiss J. Modeling dyadic effects in the associations between relational uncertainty, sexual communication, and sexual satisfaction for husbands and wives. Communic Res. 2011;38: 565–584.

[8] Kenny DA, Kashy DA, Cook WL. Dyadic data analysis. Guilford Press; 2006.

[9] Safarinejad MR, Kolahi AA, Hosseini L. The effect of the mode of delivery on the quality of life, sexual function, and sexual satisfaction in primiparous women and their husbands. J Sex Med. 2009;6: 1645–1667. 10.1111/j.1743-6109.2009.01232.x 19473472

[10] Tuinman MA, Fleer J, Sleijfer DT, Hoekstra HJ, Hoekstra-Weebers JEHM. Marital and sexual satisfaction in testicular cancer survivors and their spouses. Support Care Cancer. 2005;13: 540–548. 10.1007/s00520-004-0758-3 15660224

[11] Rehman US, Rellini AH, Fallis E. The importance of sexual self-disclosure to sexual satisfaction and functioning in committed relationships. J Sex Med. 2011;8: 3108–3115. 10.1111/j.1743-6109.2011.02439.x 21883943

[12] Mcnulty JK. Body image and marital satisfaction: Evidence for the mediating role of sexual frequency and sexual satisfaction. J Fam Psychol. 2010;24: 156–164. 10.1037/a0019063 20438191 PMC2864925

[13] Smith AMA, Patrick K, Heywood W, Pitts MK, Richters J, Shelley JM, et al Body mass index, sexual difficulties and sexual satisfaction among people in regular heterosexual relationships: A population-based study. Intern Med J. 2012;42: 641–651. 10.1111/j.1445-5994.2011.02597.x 21981105

[14] Dundon CM, Rellini AH. More than sexual function: Predictors of sexual satisfaction in a sample of women age 40–70. J Sex Med. 2010;7: 896–904. 10.1111/j.1743-6109.2009.01557.x 19889146

[15] Rosen RC, Heiman JR, Long JS, Fisher WA, Sand MS. Men with sexual problems and their partners: Findings from the International Survey of Relationships. Arch Sex Behav. 2016;45: 159–173. 10.1007/s10508-015-0568-3 26228991

[16] Hurlbert DF. A comparative study using orgasm consistency training in the treatment of women reporting hypoactive sexual desire. J Sex Marital Ther. 1993;19: 41–55. 10.1080/00926239308404887 8468709

[17] Althof SE, Buvat J, Gutkin SW, Belger M, Stothard DR, Fugl-Meyer AR. Sexual satisfaction in men with erectile dysfunction: Correlates and potential predictors. J Sex Med. 2010;7: 203–215. 10.1111/j.1743-6109.2009.01554.x 19845846

[18] Rosen RC, Brown C, Heiman JR, Leiblum SR, Ferguson D. The Female Sexual Function Index (FSFI): A multidimensional self-report instrument for the assessment of female sexual function. J Sex Martial Ther. 2000; 191–208. 10.1080/009262300278597 10782451

[19] Rosen RC, Riley A, Wagner G, Osterloh IH, Kirkpatrick J, Mishra A. The international index of erectile function (IIEF): A multidimensional scale for assessment of erectile dysfunction. Urology. 1997;49: 822–830. 9187685 10.1016/s0090-4295(97)00238-0

[20] Forbes MK, Baillie AJ, Schniering CA. Critical flaws in the Female Sexual Function Index and the International Index of Erectile Function. J Sex Res. 2014;51: 485–491. 10.1080/00224499.2013.876607 24826876

[21] American Psychiatric Association. Diagnostic and statistical manual of mental disorders. 5th ed. Washington, DC: American Psychiatric Association; 2013.

[22] Schoenfeld EA, Loving TJ, Pope MT, Huston TL, Štulhofer A. Does sex really matter? Examining the connections between spouses’ nonsexual behaviors, sexual frequency, sexual satisfaction, and marital satisfaction. Arch Sex Behav. 2016; 10.1007/s10508-015-0672-4 26732606

[23] Willoughby BJ, Farero A, Busby D. Exploring the effects of sexual desire discrepancy among married couples. Arch Sex Behav. 2014;43: 551–562. 10.1007/s10508-013-0181-2 24045904

[24] Byers ES. Relationship Satisfaction and Sexual Satisfaction: A longitudinal study of individuals in long-term relationships. J Sex Res. 2016;42: 113–118. 10.1080/00224490509552264 16123841

[25] Davies S, Katz J, Jackson JL. Sexual desire discrepancies: Effects on sexual and relationship satisfaction in heterosexual dating couples. Arch Sex Behav. 1999;28: 553–567. 10650441 10.1023/a:1018721417683

[26] Byers ES, Heinlein L. Predicting initiations and refusals of sexual activities in married and cohabiting heterosexual couples. J Sex Res. 1989;26: 210–231.

[27] Lau JTF, Yang X, Wang Q, Cheng Y, Tsui HY, Mui LWH, et al Gender power and marital relationship as predictors of sexual dysfunction and sexual satisfaction among young married couples in rural China: A population-based study. Urology. 2006;67: 579–585. 10.1016/j.urology.2005.09.039 16527583

[28] Byers ES. Beyond the birds and the bees and was it good for you: Thirty years of research on sexual communication. Can Psychol. 2011;52: 20–28.

[29] Penke L, Asendorpf JB. Beyond global sociosexual orientations: A more differentiated look at sociosexuality and its effects on courtship and romantic relationships. J Pers Soc Psychol. 2008;95: 1113 10.1037/0022-3514.95.5.1113 18954197

[30] Simpson J, Gangestad SW. Individual differences in sociosexuality: Evidence for convergent and discriminant validity. J Pers Soc Psychol. 1991;60: 870–883. 1865325 10.1037//0022-3514.60.6.870

[31] Hurlbert DF, Whittaker KE. The role of masturbation in marital and sexual satisfaction: A comparative study of female masturbators and nonmasturbators. J Sex Educ Ther. 1991;17: 272–282.

[32] Brody S, Costa RM. Satisfaction (sexual, life, relationship, and mental health) is associated directly with penile-vaginal intercourse, but inversely with other sexual behavior frequencies. J Sex Med. 2009;6: 1947–1954. 10.1111/j.1743-6109.2009.01303.x 19453891

[33] Call V, Sprecher S, Schwartz P. The incidence and frequency of martital sex in a national sample. J Marriage Fam. 1995;57: 639–652.

[34] Laumann EO, Nicolosi A, Glasser DB, Paik A, Gingell C, Moreira E, et al Sexual problems among women and men aged 40–80 y: Prevalence and correlates identified in the Global Study of Sexual Attitudes and Behaviors. Int J Impot Res. 2005;17: 39–57 10.1038/sj.ijir.3901250 15215881

[35] Liu C. Does quality of marital sex decline with duration? Arch Sex Behav. 2003;32: 55–60. 12597272 10.1023/a:1021893329377

[36] Heiman JR, Long JS, Smith SN, Fisher WA, Sand MS, Rosen RC. Sexual satisfaction and relationship happiness in midlife and older couples in five countries. Arch Sex Behav. 2011;40: 741–753. 10.1007/s10508-010-9703-3 21267644

[37] Shin DC, Johnson DM. Avowed happiness as an overall assessment of the quality of life. Soc Indic Res. 1978;5: 475–492.

[38] Arrindell WA, Heesink J, Feij JA. The Satisfaction with Life Scale (SWLS): Appraisal with 1700 healthy young adults in The Netherlands. Pers Individ Dif. 1999;26: 815–826.

[39] Pavot W, Diener E. Review of the Satisfaction With Life Scale. Psychol Assess. 1993;5: 164–172.

[40] Fugl-Meyer AR, Branholm I-B, Fugl-Meyer KS. Happiness and domain-specific life satisfaction in adult northern Swedes. Clin Rehabil. 1991;5: 25–33.

[41] Rainer H, Smith I. Education, communication and wellbeing: An application to sexual satisfaction. Kyklos. 2012;65: 581–598.

[42] Sánchez-Fuentes MM, Salinas JM, Sierra JC. Use of an ecological model to study sexual satisfaction in a heterosexual spanish sample. Arch Sex Behav. 2016; 10.1007/s10508-016-0703-9 26969318

[43] Molarius A, Berglund K, Eriksson C, Eriksson HG, Linden-Bostrom M, Nordstrom E, et al Mental health symptoms in relation to socio-economic conditions and lifestyle factors–a population-based study in Sweden. BMC Public Health. 2009;9: 302 10.1186/1471-2458-9-302 19695085 PMC2736164

[44] Adler NE, Ostrove JM. Socioeconomic status and health: What we know and what we don’t. Ann N Y Acad Sci. 1999;896: 3–15. 10681884 10.1111/j.1749-6632.1999.tb08101.x

[45] Lee D, Nazroo J, O’Connor D, Blake M, Pendleton N. Sexual health and well-being among older men and women in England: Findings from the English Longitudinal Study of Ageing. Arch Sex Behav. 2016;45: 133–144. 10.1007/s10508-014-0465-1 25624001

[46] Wiegel M, Meston C, Rosen RC. The Female Sexual Function Index (FSFI): Cross-validation and development of clinical cutoff scores. J Sex Marital Ther. 2005;31: 1–20. 10.1080/00926230590475206 15841702

[47] Berner MM, Kriston L, Rohde A. Überprüfung der Gültigkeit und Zuverlässigkeit des deutschen Female Sexual Function Index [Inspection of the validity and reliability of the German Female Sexual Function Index] (FSFI-d). Geburtsh Frauenheilk. 2004;64: 293–303.

[48] Wiltink J, Hauck EW, Phädayanon M, Weidner W, Beutel ME. Validation of the German version of the International Index of Erectile Function (IIEF) in patients with erectile dysfunction, Peyronie’s disease and controls. Int J Impot Res. 2003;15: 192–197. 10.1038/sj.ijir.3900997 12904805

[49] Rosen RC, Cappelleri JC, Gendrano N. The International Index of Erectile Function (IIEF): A state-of-the-science review. Int J Impot Res. 2002;14: 226–244. 10.1038/sj.ijir.3900857 12152111

[50] Catania JA, Dolcini MM, Coates TJ, Kegeles SM, The S, Nov N. Predictors of condom use and multiple partnered sex among sexually-active adolescent women: Implications for AIDS-related health interventions. J Sex Res. 1989;26: 514–524.

[51] Choi K, Catania J, Dolcini M. Extramarital sex and HIV risk behavior among US adults: results from the National AIDS Behavioral Survey. Am J Public Health. 1994;84: 2003–2008. 7998648 10.2105/ajph.84.12.2003 PMC1615405

[52] Diener ED, Emmons RA, Larsen RJ, Griffin S. The Satisfaction with Life Scale. J Pers Assess. 1985;49: 71–75. 10.1207/s15327752jpa4901_13 16367493

[53] Pavot W, Diener E. The Satisfaction With Life Scale and the emerging construct of life satisfaction. J Posit Psychol. 2008;3: 137–152

[54] Raudenbush SW, Bryk AS. Hierarchical linear models: Applications and data analysis methods. Sage; 2002.

[55] Cook W, Kenny D. The Actor-Partner Interdependence Model: A model of bidirectional effects in developmental studies. Int J Behav Dev. 2005;29: 101–109.

[56] R Development Core Team. R: A language and environment for statistical computing. Vienna, Austria: R Foundation for Statistical Computing 2010 Available: http://www.r-project.org

[57] Kenny DA. An interactive tool for the estimation and testing the Actor-Partner Interdependence Model using multilevel modeling. 2015. Available: https://davidakenny.shinyapps.io/APIM_MM/

[58] Cohen J. Statistical Power Analysis for the Behavioral Sciences. 2nd edn. Hillsdale, New Jersey: L. Erlbaum; 1988.

[59] Mark KP, Murray SH. Gender differences in desire discrepancy as a predictor of sexual and relationship satisfaction in a college sample of heterosexual romantic relationships. J Sex Marital Ther. 2012;38: 198–215. 10.1080/0092623X.2011.606877 22390532

[60] Schnarch D. Desire problems: A systemic perspective In: Binik YM, Hall KSK, editors. Principles and practice of sex therapy. 3rd ed. New York, NY: Guilford; 2000 pp. 17–56.

[61] Coleman E. Masturbation as a means of achieving sexual health. J Psychol Human Sex. 2003;14: 5–16.

[62] Kaestle CE, Allen KR. The role of masturbation in healthy sexual development: Perceptions of young adults. Arch Sex Behav. 2011;40: 983–994. 10.1007/s10508-010-9722-0 21293916

[63] Starrels ME. Husbands’ involvement in female gender-typed household chores. Sex Roles. 1994;31: 473–491

[64] Wiederman MW. Volunteer bias in sexuality research using college student participants. J Sex Res. 1999;36: 59–66.

