# Supplementary figures and images for: Satisfaction guaranteed? How individual, partner, and relationship factors impact sexual satisfaction within partnerships
Source: PLoS One. 2017 Feb 23;12(2):e0172855. doi: 10.1371/journal.pone.0172855 (PMC5322929; doi:10.1371/journal.pone.0172855)

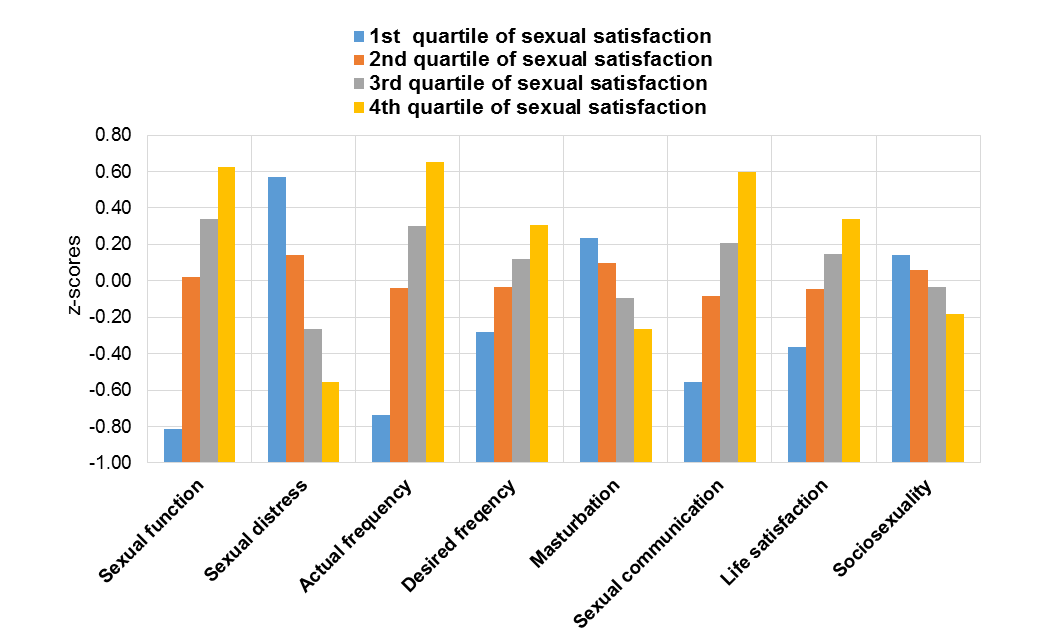

Supplement: S1 Fig — (TIF) [file pone.0172855.s002.tif]
