# Supplementary material for: Satisfaction guaranteed? How individual, partner, and relationship factors impact sexual satisfaction within partnerships
Source: PLoS One. 2017 Feb 23;12(2):e0172855. doi: 10.1371/journal.pone.0172855 (PMC5322929; doi:10.1371/journal.pone.0172855)
Supplement: S1 Table — (DOCX) [file pone.0172855.s003.docx]

| Supporting information S1  *Bivariate correlations between the actor-, partner-, relationship-related predictors, and sexual satisfaction* | | | | | | | | | | | | | | | | | | | | | | | | | |
| --- | --- | --- | --- | --- | --- | --- | --- | --- | --- | --- | --- | --- | --- | --- | --- | --- | --- | --- | --- | --- | --- | --- | --- | --- | --- |
|  |  |  | men | | | | | | | | | | | | | | | | | | | | | | |
|  |  |  | 1 | 2 | 3 | 4 | 5 | 6 | 7 | 8 | 9 | 10 | 11 | 12 | 13 | 14 | 15 | 16 | 17 | 18 | 19 | 20 | 21 | 22 | 23 |
| Sexual satisfaction (A) | 1 | women | 1 | .55‡ | .61‡ | .55‡ | -.45‡ | -.27‡ | .58‡ | -.53‡ | -.37‡ | -.30‡ | .45‡ | -.14‡ | .00 | -.16‡ | -.05 | -.14‡ | -.14‡ | -.13‡ | .25‡ | .20‡ | .03 | -.01 | .02 |
| Sexual satisfaction (P) | 2 |  | .55‡ | 1 | .44‡ | .56‡ | -.32‡ | -.41‡ | .46‡ | -.31‡ | -.48‡ | -.16‡ | .27‡ | -.09† | -.11† | -.10† | -.18‡ | -.09† | -.07* | -.06 | .20‡ | .31‡ | .03 | .01 | .01 |
| Sexual function (A) | 3 |  | .57‡ | .55‡ | 1 | .70‡ | -.45‡ | -.20‡ | .65‡ | -.34‡ | -.35‡ | -.17‡ | .36‡ | .12‡ | .07* | .10† | .01 | -.36‡ | -.36‡ | -.29‡ | .15‡ | .10† | .08* | .05 | -.03 |
| Sexual function (P) | 4 |  | .43‡ | .61‡ | .70‡ | 1 | -.27‡ | -.30‡ | .59‡ | -.34‡ | -.35‡ | -.20‡ | .32‡ | .05 | .09* | .01 | .06 | -.27‡ | -.26‡ | -.24‡ | .10† | .21‡ | .05 | .01 | .01 |
| Sexual distress (A) | 5 |  | -.42‡ | -.27‡ | -.31‡ | -.21‡ | 1 | .24‡ | -.32‡ | .24‡ | .16‡ | .09† | -.25‡ | .02 | -.04 | -.04 | .01 | .18‡ | .18‡ | .13‡ | -.22‡ | -.13‡ | -.14‡ | -.02 | .01 |
| Sexual distress (P) | 6 |  | -.32‡ | -.46‡ | -.28‡ | -.46‡ | .25‡ | 1 | -.18‡ | .17‡ | .17‡ | .14‡ | -.16‡ | .04 | .00 | .01 | -.02 | .05 | .03 | .04 | -.17‡ | -.29‡ | -.16‡ | .01 | -.01 |
| Sexual frequency (D) | 7 |  | .52‡ | .55‡ | .67‡ | .64‡ | -.18‡ | -.26‡ | 1 | -.54‡ | -.30‡ | -.20‡ | .38‡ | .05 | .07* | -.01 | .03 | -.25‡ | -.25‡ | -.28‡ | .12‡ | .08† | .00 | -.02 | .05 |
| Sexual discrepancy (A) | 8 |  | -.49‡ | -.37‡ | -.35‡ | -.36‡ | .18‡ | .17‡ | -.42‡ | 1 | .21‡ | .29‡ | -.28‡ | .19‡ | .03 | .26‡ | .07* | -.07* | -.08* | .01 | -.16‡ | -.09† | .01 | .03 | -.06 |
| Sexual discrepancy (P) | 9 |  | -.31‡ | -.53‡ | -.34‡ | -.34‡ | .18‡ | .25‡ | -.35‡ | .22‡ | 1 | .05 | -.13‡ | .06 | .12‡ | .11† | .21‡ | .01 | .01 | -.03 | -.11† | -.13‡ | -.04 | -.04 | .02 |
| Sexual initiative (D) | 10 |  | -.15‡ | -.14‡ | -.15‡ | -.04 | .21‡ | .06 | -.10† | .12† | .14‡ | 1 | -.26‡ | .07 | -.04 | .08† | -.02 | .00 | .00 | .07* | -.02 | -.06 | .00 | .01 | -.04 |
| Sexual communication (A) | 11 |  | .41‡ | .34‡ | .41‡ | .33‡ | -.28‡ | -.22‡ | .36‡ | -.20‡ | -.16‡ | -.20‡ | 1 | -.06 | .14‡ | -.10† | .04 | -.14‡ | -.16‡ | -.17‡ | .15‡ | .09† | -.03 | .00 | .00 |
| Sociosexual orientation (A) | 12 |  | -.11† | .00 | .09† | .07* | .00 | -.04 | .03 | .11† | .05 | -.03 | .04 | 1 | .39‡ | .43‡ | .29‡ | -.18‡ | -.19‡ | -.25‡ | -.11† | -.04 | .12‡ | .02 | .05 |
| Sociosexual orientation (P) | 13 |  | -.10† | -.15‡ | .04 | .11† | .04 | .03 | .04 | .08* | .20‡ | .04 | .07* | .38‡ | 1 | .22‡ | .41‡ | -.21‡ | -.23‡ | -.32‡ | -.03 | -.06 | .02 | -.02 | .09† |
| Masturbation (A) | 14 |  | -.17‡ | -.05 | .06 | .03 | .00 | .01 | -.03 | .22‡ | .09* | .00 | -.02 | .41‡ | .30‡ | 1 | .28‡ | -.38‡ | -.37‡ | -.33‡ | -.12‡ | -.04 | .13‡ | -.02 | .03 |
| Masturbation (P) | 15 |  | -.11† | -.16‡ | .01 | .09* | .02 | -.03 | -.01 | .11† | .27‡ | .03 | .06 | .22‡ | .44‡ | .28‡ | 1 | -.19‡ | -.22‡ | -.24‡ | -.09† | -.10† | .07* | .00 | .05 |
| Age (A) | 16 |  | -.08† | -.15‡ | -.27‡ | -.37‡ | .04 | .19‡ | -.29‡ | .02 | -.07* | .02 | -.22‡ | -.23‡ | -.18‡ | -.21‡ | -.36‡ | 1 | .92‡ | .73‡ | .09† | .01 | -.02 | .09† | -.01 |
| Age (P) | 17 |  | -.10† | -.16‡ | -.28‡ | -.37‡ | .06 | .18‡ | -.30‡ | .02 | -.07* | .01 | -.22‡ | -.20‡ | -.18‡ | -.17‡ | -.36‡ | .92‡ | 1 | .77‡ | .10† | .03 | .00 | .09† | -.01 |
| Relationship duration (D) | 18 |  | -.08† | -.15‡ | -.24‡ | -.30‡ | .06 | .14‡ | -.27‡ | -.02 | .02 | .07* | -.22‡ | -.31‡ | -.24‡ | -.22‡ | -.31‡ | .78‡ | .74‡ | 1 | .09† | .06 | .02 | .07 | -.12† |
| Satisfaction with life (A) | 19 |  | .32‡ | .20‡ | .21‡ | .10† | -.30‡ | -.14‡ | .14‡ | -.14‡ | -.09† | -.11† | .26‡ | -.08* | -.05 | -.09† | -.04 | .02 | 0 | .06 | 1 | .36‡ | .24‡ | .08* | -.08* |
| Satisfaction with life (P) | 20 |  | .20‡ | .25‡ | .10† | .15‡ | -.18‡ | -.23‡ | .09† | -.11† | -.16‡ | -.08* | .12† | -.05 | -.13‡ | -.09† | -.11† | .09† | .07* | .09† | .36‡ | 1 | .24‡ | .01 | -.03 |
| Household income (D) | 21 |  | .03 | .02 | .07 | .08* | -.21‡ | -.12‡ | .00 | -.03 | .03 | -.02 | .03 | .05 | .05 | .07* | .10† | -.04 | -.05 | -.01 | .24‡ | .22‡ | 1 | .12† | -.14‡ |
| % of household income (A) | 22 |  | .01 | .02 | .01 | -.02 | -.01 | .01 | -.01 | .01 | -.06 | -.07* | -.02 | .08* | .05 | .04 | .03 | .00 | 0 | -.11† | -.04 | -.10† | -.11† | 1 | -.61‡ |
| % of household income (A) | 23 |  | .00 | -.02 | .01 | .03 | .02 | -.02 | .02 | -.04 | .03 | .03 | -.05 | -.01 | .03 | .02 | .00 | .10† | .09† | .08* | .00 | .07* | .06 | -.59‡ | 1 |

* *p* < .05, † *p* < .01, ‡ *p* < .001

*Note.* A = actor variable, P = partner variable, D = between dyads variable
