# Supplementary material for: Satisfaction guaranteed? How individual, partner, and relationship factors impact sexual satisfaction within partnerships
Source: PLoS One. 2017 Feb 23;12(2):e0172855. doi: 10.1371/journal.pone.0172855 (PMC5322929; doi:10.1371/journal.pone.0172855)
Supplement: S2 Table — (DOCX) [file pone.0172855.s004.docx]

| S3  *Overall effect estimates for the actor-partner interdependence model for sexual satisfaction* | | | | | | | | | | | | |
| --- | --- | --- | --- | --- | --- | --- | --- | --- | --- | --- | --- | --- |
| Variable | Effect | Estimate | CI (95 %) | | | | *p* | | β | | *r* | |
| Sexual satisfaction | Intercept | 70.13 | 68.63 | – | 71.64 | < .001 | |  | |  | |  |
| Sexual function | Actor | 7.77 | 6.37 | – | 9.16 | < .001 | | .28 | | .27 | |  |
|  | Partner | 0.91 | -0.44 | – | 2.26 | .190 | | .03 | | .03 | |  |
| Sexual distress | Actor | -0.15 | -0.19 | – | -0.12 | < .001 | | -.17 | | -.21 | |  |
|  | Partner | -0.07 | -0.10 | – | -0.03 | < .001 | | -.07 | | -.10 | |  |
| Frequency of sexual activity | | 2.23 | 1.00 | – | 3.45 | < .001 | | .09 | | .18 | |  |
| Desire discrepancy | Actor | -5.71 | -7.19 | – | -4.24 | < .001 | | -.18 | | -.20 | |  |
|  | Partner | -2.22 | -3.56 | – | -0.87 | .001 | | -.07 | | -.08 | |  |
| Sexual initiative |  | -2.59 | -4.10 | – | -1.08 | < .001 | | -.07 | | -.09 | |  |
| Sexual communication |  | 3.22 | 2.32 | – | 4.12 | < .001 | | .15 | | .19 | |  |
| Sociosexual orientation | Actor | -3.15 | -4.43 | – | -1.86 | < .001 | | -.11 | | -.13 | |  |
|  | Partner | 0.23 | -1.03 | – | 1.49 | .720 | | .01 | | .01 | |  |
| Masturbation | Actor | -1.37 | -2.17 | – | -0.56 | < .001 | | -.08 | | -.09 | |  |
|  | Partner | 0.26 | -0.51 | – | 1.03 | .510 | | .02 | | .01 | |  |
| Age | Actor | 0.11 | -0.05 | – | 0.28 | .190 | | .06 | | .04 | |  |
|  | Partner | -0.18 | -0.34 | – | -0.01 | .030 | | -.09 | | -.05 | |  |
| Relationship duration |  | 0.10 | -0.02 | – | 0.22 | .110 | | .05 | | .05 | |  |
| Life satisfaction | Actor | 2.20 | 1.36 | – | 3.03 | < .001 | | .10 | | .14 | |  |
|  | Partner | 0.72 | -0.10 | – | 1.54 | .090 | | .03 | | .05 | |  |
| Household income |  | -0.62 | -1.04 | – | -0.20 | .004 | | -.06 | | -.09 | |  |
| % of household income | Actor | 0.04 | -0.02 | – | 0.09 | .199 | | .04 | | .04 | |  |
|  | Partner | 0.05 | -0.00 | – | 0.11 | .064 | | .06 | | .56 | |  |
