# Supplementary material for: Satisfaction guaranteed? How individual, partner, and relationship factors impact sexual satisfaction within partnerships
Source: PLoS One. 2017 Feb 23;12(2):e0172855. doi: 10.1371/journal.pone.0172855 (PMC5322929; doi:10.1371/journal.pone.0172855)
Supplement: S1 Text — (DOCX) [file pone.0172855.s005.docx]

The model used to investigate the impact of our predictors on actor’s sexual satisfaction used the following formula:

Sexual satisfaction _ij_ = β_0i_ + β_1_(Actor sexual function) _ij_ + β_2_(Partner sexual function) _ij +_ β_3_(Actor sexual distress) _ij_ + β_4_(Partner sexual distress) _ij_ + β_5_(Frequency of sexual activity) _j_ + β_6_(Actor desire discrepancy) _ij+_  β_7_(Partner desire discrepancy) _ij_ + β_8_(Sexual initiation) _j_ + β_9_(Actor masturbation) _ij_ + β_10_(Partner masturbation) _ij_ + β_11_(Actor age) _ij_ + β_12_(Partner age) _ij_ + β_13_(Relationship duration) _j_ + β_14_(Actor life satisfaction) _ij_ + β_15_(Partner life satisfaction) _ij_ + β_16_(Household income) _j_ + β_17_(Actor percentage of household income) _ij_ + β_18_(Partner percentage of household income) _ij_ + r_0i_  + ε _ij_

where sexual satisfaction _ij_ is the _i_th individual’s sexual satisfaction in dyad _j_.

In this model, β_0i_ is the individual-specific intercept, β_1_ is the individual specific slope, r_0i_ describes the random intercept and ε_ij_ indicates the residuals. In addition, interactions with gender were also investigated for all predictors.
